# As expected, based on rapamycin-like p53-mediated gerosuppression, mTOR inhibition acts as a checkpoint in p53-mediated tumor suppression

**DOI:** 10.18632/oncoscience.561

**Published:** 2022-08-30

**Authors:** Mikhail V. Blagosklonny

**Affiliations:** ^1^Roswell Park Comprehensive Cancer Center, Buffalo, NY 14263, USA

**Keywords:** senescence, geroconversion, p53, sirolimus, cancer, hyperfunction theory

Recent work by Gu and co-workers (Kon et al., published in 2021), entitled “mTOR inhibition acts as an unexpected checkpoint in p53-mediated tumor suppression”, seemingly “unexpectedly” demonstrated in mice that the ability of p53 to suppress mTOR is essential for tumor suppression early in life [1]. This actually was predicted in 2012 in the commentary entitled “Tumor suppression by p53 without apoptosis and senescence: conundrum or rapalog-like gerosuppression?” [2] [Note: rapalogs are rapamycin analogs]. The commentary [2] was written on another fascinating paper by the same senior author Gu and co-workers (Li et al.) “Tumor suppression in the absence of p53-mediated cell-cycle arrest, apoptosis, and senescence” [3].

**Figure 1:**
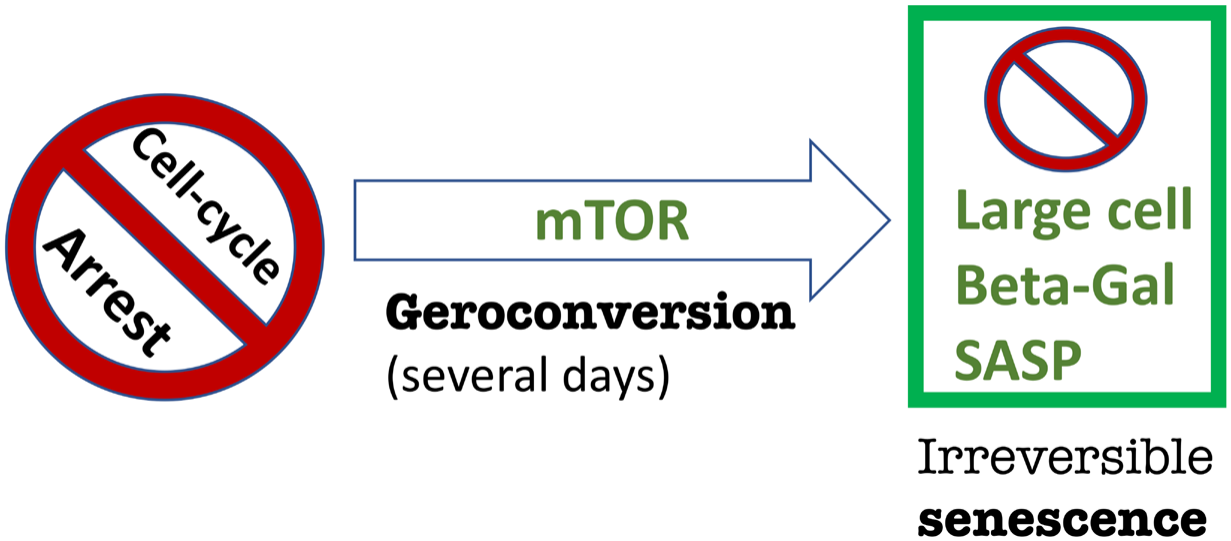
Geroconversion from cell cycle arrest to senescence. Simplified schema. In addition to mTOR, other signaling pathways such as MAPK are involved in geroconversion (not shown).

Mutant p53 (p53-3KR), constructed by Li et al., lacking all three then-known tumor-suppressing activities, still suppressed tumors [3]. To be precise, as noticed in the commentary [2], there were only two, not three, independent tumor-suppressing activities of p53 known at that time: namely, (i) apoptosis and (ii) cell-cycle arrest/senescence. Wild-type p53 does not directly induce the senescent phenotype; it induces cell-cycle arrest, which then converts to senescence (geroconversion) without any p53 assistance (Figure 1).

When the cell cycle gets arrested by any means (by p53, p21 or anything else), the arrested cell is not yet senescent at first. It will take several days (at least) in cell culture to observe senescent phenotype, including large cell morphology, Senescence-Associated Secretory Phenotype (SASP) and beta-Gal-staining (Figure 1). Geroconversion is driven by growth-promoting pathways such as mTOR and MAPK [4]. In fact, rapamycin and anything that inhibits mTOR such as serum-starvation, contact inhibition and anoxia partially suppresses geroconversion and the senescent phenotype (see for ref. [4]).

**Figure 2:**
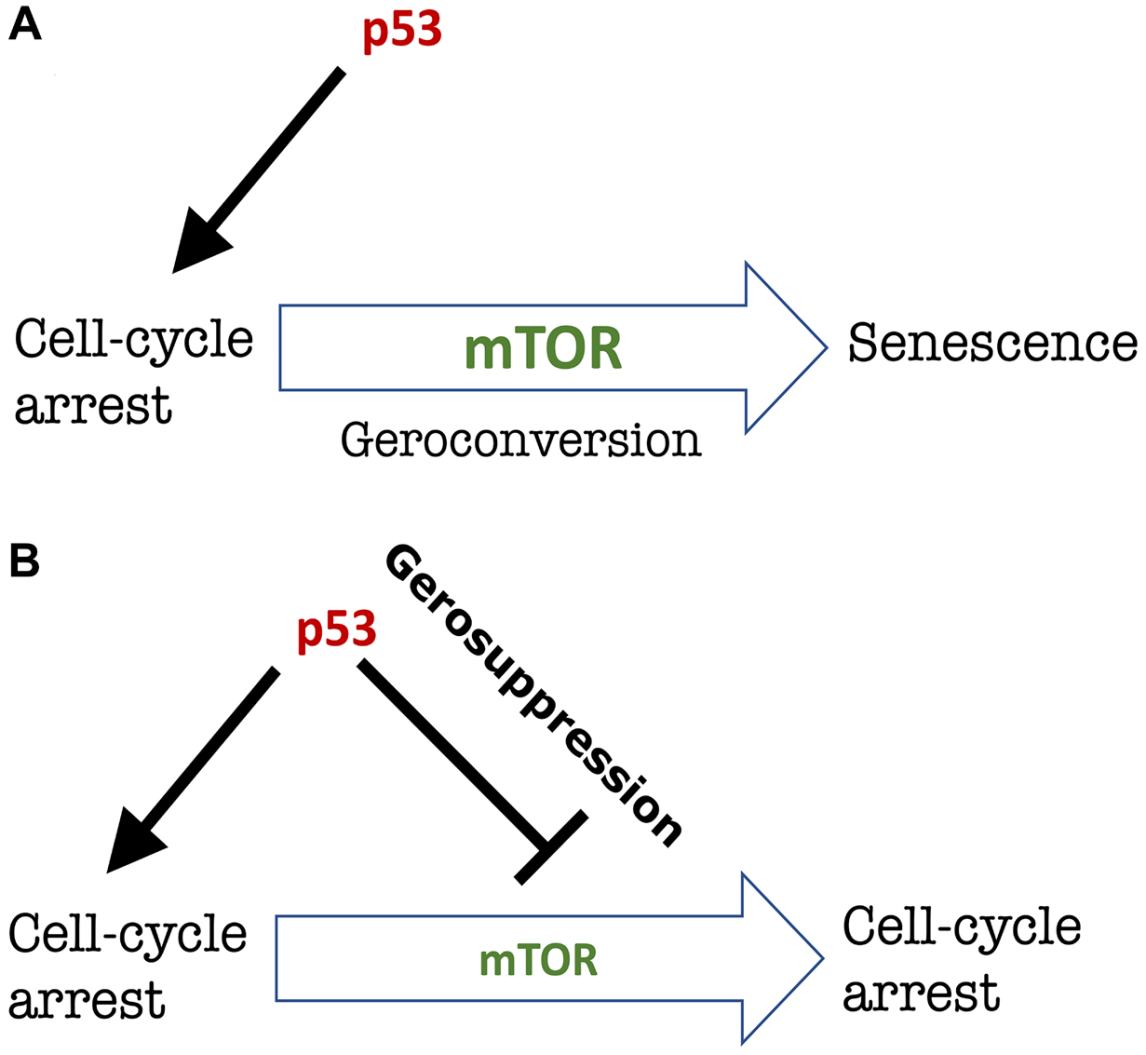
The choice between cell-cycle arrest and senescence. (**A**) p53-induced senescence. p53 induces cell-cycle arrest. mTOR then drive geroconversion to senescence [2, 5, 6]. (**B**) p53-induced gerosuppression. p53 induces cell-cycle arrest and simultaneously inhibits mTOR. This suppresses geroconversion and the cell remains arrested but not senescent [2, 5, 6]. The ability of p53 to inhibit mTOR is context-dependent as discussed [6].

Then how does p53 causes senescence? It causes cell-cycle arrest, which, in growth-factor rich cell culture, may automatically lead to a senescent phenotype (Figure 2A).

[In analogy, a key to your home seemingly has two activities unlock the door and open the door. Yet, it only unlocks the door. When the door is unlocked by the key, you (or the wind) may open the door without key. But if an altered ”mutant” key cannot unlock the door, it cannot help to open it either].

Since mutant p53 (p53-3KR) cannot cause cell-cycle arrest, it cannot cause senescence either. On another hand, p53 may inhibit senescence by inhibiting mTOR-driven geroconversion [5, 6]. p53 inhibits mTOR [5–8]. When p53 causes quiescence (reversible arrest), it does so by inhibiting geroconversion (Figure 2B). The experimental confirmation is described [5] and discussed elsewhere [2, 9], so I will not discuss it here.

In agreement with *in vitro* results, it was shown that p53-null mice have increased mTOR activity [10], and that observation was confirmed by Kon et al., [1]. Rapamycin also delays cancer and increases lifespan in p53+/− mice [11, 12].

Kon et al., constructed a p53 mutant (p53-5KR) that is unable to inhibit mTOR [1]. Kon et al., showed that loss of mTOR inhibition led to inability to suppress tumors early in life. This defect was mitigated/reversed by treatment with rapamycin, further supporting the role of mTOR-inhibition in a cancer checkpoint [1]. As shown previously, rapamycin delayed tumorigenesis and extended lifespan in p53-null mice [13], an observation also confirmed by Kon et al., [1].

**Figure 3:**
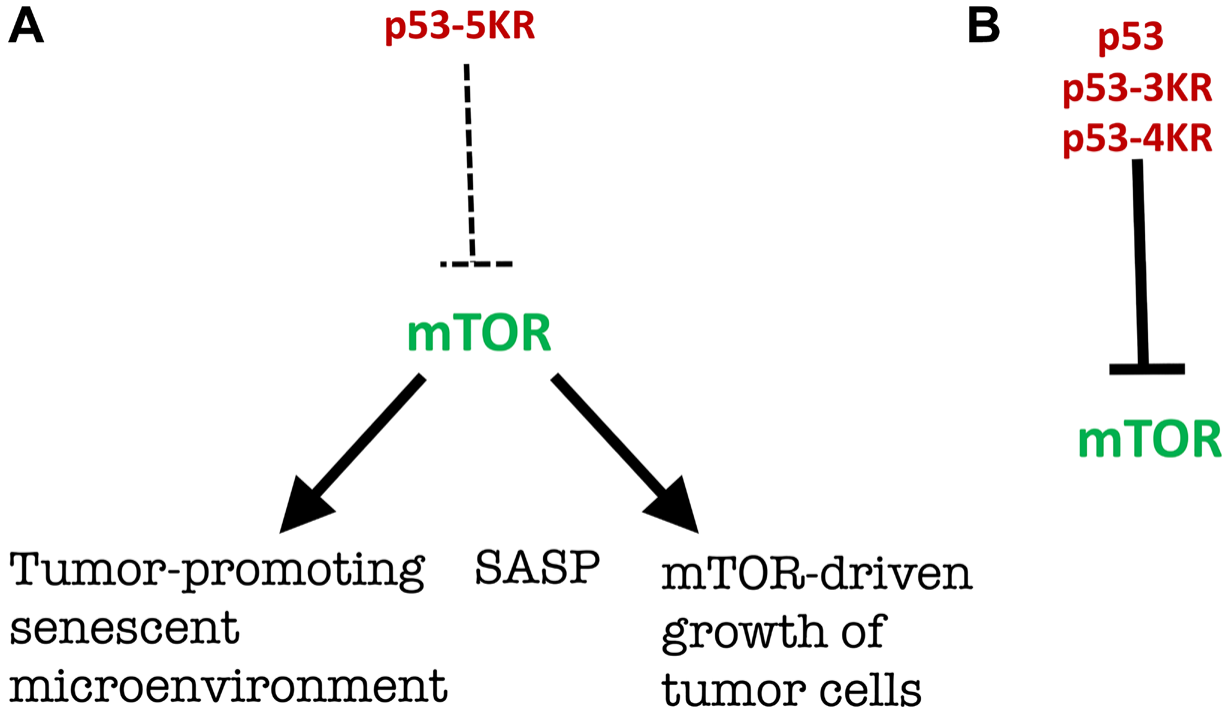
Rapamycin-like gerosuppression by p53. (**A**) p53-5KD does not inhibit mTOR, mTOR is hyperactivated and favors the senescent microenvironment and SASP that promotes tumorigenesis. Also hyperactivated mTOR directly stimulates growth of cancer cells. (**B**) Wt p53, p53-3KD and p53-4KD prevent mTOR hyper-activation.

As anticipated in 2012, in the absence of rapamycin-like p53-mediated gerosuppression, mTOR favors a senescent microenvironment [2], associated with cancer promotion [14–17]. SASP is one of the mechanisms of tumor-promotion by senescent cells and is inhibited by both rapamycin and p53 [12, 18–20]. Rapamycin decreases the ability of microenvironment and tumor stroma to promote cancer growth [12, 15, 16]. Also, mTOR may directly increase pre-cancer/cancer cell growth (Figure 3).

To keep the focus on the mTOR story, which was unexpected for Kan et al., [1], I did not discuss the fourth anti-cancer activity of p53 discovered by Gu and co-workers: ferroptosis [1, 21, 22]. I still must mention this fascinating story because p53-5KD should be compared with p53-4KD, not with p53-3KD. Mutant p53-4KR, which lacks the ability to undergo p53-mediated cell cycle arrest/senescence, apoptosis, and ferroptosis, retains the ability to inhibit mTOR activity, while this activity is completely abolished in p53-5KR [1]. This work by Gu and co-workers adds a fourth activity (ferroptosis) [1, 21, 22] to three anti-cancer activities proposed in 2012 [2]: cycle arrest, apoptosis, and rapamycin-like gerosuppression or, in simple words, mTOR inhibition [2].
